# Potentially Hygroreceptive Sensilla on the Anal Stylus of the Glassy-Winged Sharpshooter, *Homalodisca vitripennis*

**DOI:** 10.1673/031.008.5801

**Published:** 2008-10-13

**Authors:** Natalie A. Hummel, Walter S. Leal, Frank G. Zalom

**Affiliations:** ^1^ Department of Entomology, Louisiana State University Agricultural Center, 404 Life Sciences Building, Baton Rouge, LA 70803, USA; ^2^ Department of Entomology, University of California, One Shields Avenue, Davis, CA 95616, USA

**Keywords:** coeloconic sensilla, stylogram, hygroreceptor, leafhopper

## Abstract

This study begins to elucidate the cues and mechanisms by which the glassy-winged sharpshooter, *Homalodisca vitripennis* (Germar) (Hemiptera: Cicadellidae), select host plants for feeding and oviposition. The electrophysiological response of the anal styli of male and female *H. vitripennis* to water vapor was examined using a modified electroantennography (EAG) device (stylogram). A strong electrophysiological response of the anal stylus to water vapor was found. Scanning electron microscopic examination of the anal stylus revealed the presence of long mechanosensory hairs, many small coeloconic-type sensilla, and masses of secretory granules termed brochosomes. Each coeloconic sensillum is located in a pit from which protrude finger-like projections. The pit is often blocked by masses of brochosomes and an unidentified dense material. Based on the electrophysiological response of the anal stylus to water vapor, we hypothesize that the coeloconic sensilla on the stylus may be hygroreceptors. *H. vitripennis* are xylem feeders and may use the sensilla to assist in host selection for the purpose of feeding or oviposition based on detected plant water status. Furthermore, *H. vitripennis* oviposit into the leaf epidermis, and may use these sensilla to evaluate moisture content to determine host suitability for both oviposition and subsequent feeding of emerged progeny. Understanding the cues and underlying mechanisms of host selection is an important consideration for predicting the movement of *H. vitripennis* between crops and disease epidemiology.

## Introduction

The glassy-winged sharpshooter, *Homalodisca vitnpennis* (Germar) (Hemiptera: Cicadellidae), was first detected in southern California in 1989 (Sorenson and Gill 1996), and has spread to fifteen counties in California (CDFA 2006). *H. vitripennis* is distributed throughout the southern United States and into South America (Young 1958; Turner and Pollard 1959) and may spread to the grape-growing regions of northern Argentina and southern of Brazil (Peterson et al. 2003). It has recently invaded the islands of Hawaii and Tahiti (Hoddle 2004). It is considered the most significant insect pest threatening the California grape industry (Purcell 1999; Purcell and Saunders 1999). *H. vitripennis* is of greatest concern as a vector of the bacterium *Xylella fastidiosa* (Wells et al. 1987) that causes Pierce's disease of grapes (Alderz and Hopkins 1979; Turner 1949; Hewitt et al. 1946), almond leaf scorch and oleander leaf scorch (Costa et al. 2000; Davis et al. 1980). The bacterium is vectored to uninfected hosts when the insect moves between host plants and feeds on their xylem fluids (Purcell and Saunders 1999). Insects of many species choose host plants based on a combination of visual and olfactory cues. The visual and olfactory cues that attract *H. vitripennis* to a given host species are unknown. Migration of *H. vitripennis* populations between oranges and lemons as vegetative flushes occur has been observed. This movement has been correlated with seasonally fluctuating amino acid levels in the xylem fluid of the host species (Bi et al. 2005). Field observations in central and southern California also indicate that *H. vitripennis* move *en masse* from one host to another in synchrony with irrigation schedules (MW Johnson and RL Groves, personal commmunication).

*H. vitripennis* feed primarily on xylem sap drawn from xylem conducting vessels (Leopold et al. 2003), extracting 100–300 times their body weight per day (Brodbeck et al. 1993). The anal stylus is oriented away from the body during feeding allowing watery excrement to be released away from the body. This posture may also allow the anal stylus to function as a sensory structure for the purpose of detecting chemical and environmental cues. Researchers have not successfully identified any chemical, or physical cues by which *H. vitripennis* select specific host plants for feeding and oviposition in a complex landscape.

The objectives of this study were to determine if there exists an electrophysiological response of the anal stylus of *H. vitripennis* to water vapor, and to describe the external morphology of the putative hygroreceptive sensilla located on the anal stylus. This electrophysiological response to water vapor may indicate a mechanism by which *H. vitripennis* are able to orient toward and select hosts with the desired specific water status. Understanding mechanisms by which *H. vitripennis* select host plants has significant implications for disease transmission epidemiology.

## Materials and Methods

### Stylograms

This study utilized a method equivalent to electroantennogram to record the electrophysiological response of the anal stylus to water vapor. This method is referred to as a stylogram. Five female and five male *H. vitripennis* were collected from citrus hosts at University of California, Riverside Agricultural Operations and maintained in mesh cages with fresh plant material before dissection. The anal stylus was dissected, removing it at the point where it joined the posterior region of the abdomen. The stylus was mounted on the electrode of a 10× EAG Preamplifier (Syntech, www.syntech.nl) with the tip of the stylus connecting to the recording electrode and the bottom contacting the indifferent electode. Electric contact was made with Spectra 360 electrode gel (Parker Laboratories, www.parkerlabs.com). The signal from the electrode was fed onto a Data Acquisition Interface, IDAC-232 (Syntech) and then routed to a Dell Latitude D 600 computer. The preparation at room temperature (25 °C) was continuously flushed by a charcoal-filtered air stream (300 ml/min) delivered by a CS-05 stimulus controller (Syntech) through a Teflon coated tube (7 mm, i.d) ending near (3–4 mm) the preparation. The tube had a 2 mm hole 6 cm away from the preparation for insertion of a stimulus pipette. The air stream was diverted through the stimulus pipette for 0.5 s with compensation to maintain a constant flow. For the treated assay, a filter paper strip (0.5 × 2 cm; Whatman #1 qualitative) saturated with deionized water was placed in a 14.5 cm-long Pasteur pipette. The control assay was a dry filter paper strip placed in a 14.5 cm-long Pasteur pipette.

### Scanning Electron Micrographs

*H. vitripennis* were collected from citrus at UCR Agricultural Operations and stored in 70% ethanol. Specimens were then dehydrated in a graded ethanol series, ultrasonically cleaned in 95% ethanol, soaked overnight in hexane, and dried on a Whatman No. 1 filter paper in a covered Petri dish for at least two days. Specimens were mounted on aluminum stubs (Ted Pella, Inc., www.tedpella.com) using adhesive tabs (Ted Pella, Inc.), then coated with palladium gold for ninety seconds using a sputter coater (Denton Vacuum Desk II Cold Sputter-Etch Unit, Denton Vacuum, www.dentonvacuum.com). The prepared specimens were viewed with a scanning electron microscope (S3500N SEM, Hitachi, www.hitachi-hta.com). Digital images were captured and contrast adjusted using Adobe Photoshop® (Adobe Systems Inc., www.adobe.com).

## Results

### Stylogram

**Figures 1–4.**
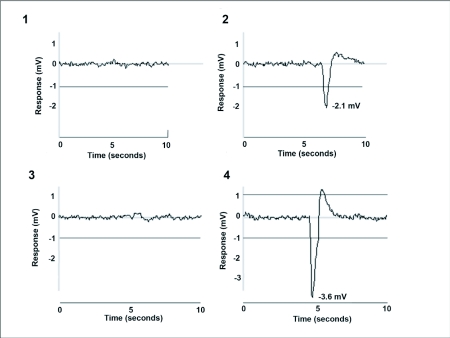
Stylograms of the response of the anal stylus of a male *Homalodisca vitripennis* to (1) a blank filter paper and (2) water vapor; a female *Homalodisca vitripennis* to (3) a blank filter paper and (4) water vapor.

The anal styli of male (Figures 1 and 2) and female (Figures 3 and 4) *H. vitripennis* both exhibited stronger electrophysiological responses to air puffed over a filter paper strip saturated with water (Figures 2 and 4) than their respective controls (Figures 1 and 3), with females giving stronger responses (7.1 ± 0.8 mV) than males (3.7 ± 0.4 mV).

### Scanning Electron Micrographs

**Figures 5–9.**
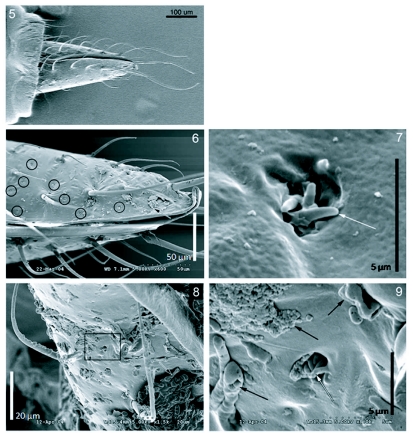
Scanning electron micrographs of the anal stylus of a male *Homalodisca vitripennis* (5, 6). A magnified image of a single potentially hygroreceptive sensillum with three finger-like projections in a pit that is located on the anal stylus (7). Scanning electron micrographs of the anal stylus of a female *Homalodisca vitripennis* (8) with a magnified image (black box) of a single potentially hygroreceptive sensillum with six finger-like projections in a pit (9). Black arrow, brochosomes; black circle, coeloconic sensilla; white arrow, finger-like projections in a coeloconic sensillum.

The anal styli of male (Figure 5) and female (Figure 8) *H. vitripennis* possess sensilla (Figure 6, circles) that may function as hygroreceptors. The anal styli of male and female *H. vitripennis* are very similar in appearance. The structure consists of a pit from which protrudes a varying number (3 to 6) of finger-like projections (Figures 7 and 9, white arrow). The pit is approximately 2.5 *µ*m in diameter and are often observed to be blocked by masses of the secretory granules termed brochosomes (Rakitov 2002) (Figures 6, 8, 9, black arrow). Based on the external anatomy, the sensilla type is most likely coeloconic. Sensilla with 3 (Figure 7) or 6 finger-like projections (Figure 9) are located on the anal styli of both male and female *H. vitripennis*.

## Discussion

Hygroreceptors have been documented in many insect families. The cockroach, *Periplaneta americana* (Altner et al. 1977) has cold-moist-dry cells in thermoreceptors and hygroreceptors. These are grooved hairs and double-walled sensilla (Altner et al. 1977). Hygroreceptors have also been described in Locusts (Waldow 1970). Hygroreceptive sensilla basiconica have been described in kissing bugs (Bernard 1974) and the larvae of many species of Lepidoptera (Dethier and Schoonhoven 1968). In *Aedes* mosquitoes hygroreceptors have the form of grooved pegs (Kellog 1970).

Insects use hygroreceptive sensilla to measure host water content and relative humidity in the environment (Dethier and Schoonhoven 1968). In caterpillars, the ability to sense water content of the host may be used as an indicator of host quality (Dethier and Schoonhoven 1968). A number of different types of sensilla have been identified on the mouthparts (Leopold et al. 2003) and the ovipositor (Hummel et al. 2006) of *H. vitripennis*. The sensilla on the anal stylus of *H. vitripennis* have similar morphology to hygroreceptors found in other taxa (Altner et al. 1983; Hull and Cribb 1997; Le Ru et al. 1995; Waldow 1970). Hygroreceptors may present an advantageous evolutionary adaptation to *H. vitripennis* as they are generally found in a warmer and drier climatic zone of the southern United States, Central and South America. The adults and nymphs feed primarily on xylem conducting vessels (Leopold et al. 2003) and females oviposit in the leaf epidermis (Turner and Pollard 1959). The ability to detect xylem flows and leaf quality could have significant implications for host selection. Field observations suggest that *H. vitripennis* move *en masse* from one host to another in synchrony with irrigation schedules (MW Johnson and RL Groves, personal communication). This movement may be mediated in part by the hygroreceptive sensilla on the anal stylus and/or the ovipositor.

Xylem feeders are characterized by a high rate of feeding when the quality of the food resource is poor. The anal stylus of *H. vitripennis* has two functions. It releases large amounts of excrement at a rapid rate and brochosomes which females deposit onto egg masses immediately after oviposition (Rakitov 2002). The location of the anal stylus and the potentially hygroreceptive sensilla dorsal to the ovipositor may also play a role in selecting a host for oviposition as they are situated in such a manner that they may be able to detect the relative water content of the host leaf after it is punctured by the ovipositor. Single-sensillum recordings and transmission electron microscopic examination of the sensilla would be necessary to establish their function and to identify sensillum type.

In conclusion, the stylograms of anal styli of both female and male *H. vitripennis* expressed a strong electrophysiological response to water vapor. This response suggests that hygroreceptive sensilla are present on the anal stylus. Scanning electron microscopic examination of the anal stylus revealed large mechanosensory trichoid sensilla and coeloconic sensilla. Based on the similar morphology of these structures to hygroreceptors previously described in the literature for other taxa (Altner et al. 1983; Hull and Cribb 1997; Le Ru et al. 1995; Waldow 1970), we conclude that the coeloconic sensilla on the anal stylus of *H. vitripennis* may be hygroreceptors. These hygroreceptors may provide sensory information used in selection of a host species for feeding and/or oviposition. Failure to find feeding or oviposition sites could result in movement between hosts that can result in the spread of *X. fastidiosa* that causes Pierce's disease of grapevines (Alderz and Hopkins 1979; Hewitt et al. 1946) and oleander leaf scorch (Costa et al. 2000). Understanding the sensory mechanisms that regulate movement between hosts could potentially be used in manipulations designed to decrease or eliminate disease spread.
